# Wastewater surveillance as an early warning system for fungal pathogens

**DOI:** 10.1371/journal.ppat.1014475

**Published:** 2026-08-07

**Authors:** Pooja Padmakumar, Adam Winrow, Pilvi Hepo-oja, Jane Usher, Edward J. Feil, Harriet C. Davidson, Tihana Bicanic, Davey L. Jones, Johanna Rhodes

**Affiliations:** 1 School of Environmental & Natural Sciences, Bangor University, Gwynedd, United Kingdom; 2 Department of Medical Microbiology, University Medical Center Utrecht, Utrecht, Netherlands; 3 Medical Research Council Centre for Medical Mycology, University of Exeter, Exeter, United Kingdom; 4 The Centre for Evolution, Department of Life Sciences, University of Bath, Bath, United Kingdom; 5 Institute of Infection & Immunity, St. George’s School of Health and Medical Sciences, City St. George’s University of London, London, United Kingdom; 6 Verily Life Sciences LLC , Dallas, United States of America; 7 School of Biosciences, University of Birmingham, Birmingham, United Kingdom; University of Maryland, Baltimore, UNITED STATES OF AMERICA

## Introduction

Wastewater-based surveillance (WBS) involves the routine molecular and microbiological analysis of municipal sewage to track pathogen circulation across communities, without relying on individual clinical testing. The COVID-19 pandemic established WBS as an effective early warning system, with SARS-CoV-2 detection preceding clinical case surges by 4–14 days. These findings prompted investment in viral and bacterial wastewater monitoring infrastructure [1–3]. In contrast, invasive fungal infections cause approximately 2.5 million deaths annually (more than tuberculosis and malaria combined) yet receive disproportionately limited research investment and public health prioritisation [4]. Hospital outbreaks caused by drug-resistant *Candida* species are increasingly common as fungi adapt to human body temperatures, healthcare environments, and antifungal pressure [4–6]. Despite the World Health Organization’s (WHO) designation of priority fungal pathogens requiring urgent surveillance innovation, this burden has not yet translated into public health action [5].

The capacity to build coordinated surveillance systems is well established, as evidenced by the global polio monitoring and antibiotic resistance monitoring in bacteria, which evolved from fragmented national initiatives into a globally coordinated infrastructure [1]. Climate change, expanding immunocompromised populations and paucity of antifungal therapeutics create ecological and clinical conditions favourable to the emergence and spread of fungal pathogens; however, the development of a coordinated surveillance strategy to detect fungal outbreaks lags behind this evolving threat [6]. Here, we examine why existing clinical surveillance frameworks fail for fungi, review evidence for *Candida* WBS, and identify the key research priorities to address this gap.

## Why is clinical surveillance for fungal pathogens structurally inadequate?

Three factors contribute to the structural inadequacy of systematic fungal surveillance. First, categorisation bias assigns fungi to a level of secondary importance. Unlike bacteria or viruses that trigger acute and clinically apparent outbreaks, fungal pathogens like *Candida* and *Aspergillus* manifest as opportunistic infections in immunocompromised populations [4]. This epidemiological pattern promotes their dismissal as complications of underlying conditions rather than primary public health threats requiring dedicated surveillance infrastructure [5,6]. Most WHO priority fungal pathogens outside *Candida* and *Aspergillus* are environmentally acquired with limited human-to-human transmission, placing them outside traditional outbreak surveillance frameworks in the UK. Mandatory *C. auris* laboratory notification in the UK was introduced in 2025, highlighting the recent development of systematic mandatory pathogenic fungal surveillance [7].

Second, fungal colonisation biology presents distinct surveillance challenges. Extended asymptomatic colonisation periods (especially in commensal organisms like *Candida*) often precede symptomatic infection in susceptible hosts, sometimes persisting from months to years. Colonised individuals remain clinically well but continuously shed viable organisms. Nosocomial *Candida* strains are transmissible and shed from skin and gut directly into sewage and the wider environment. *Aspergillus*, in contrast, is acquired through inhalation of environmental conidia and not transmitted via gastrointestinal routes into water. Existing clinical monitoring therefore detects neither until invasive disease occurs, capturing only the minority of colonised individuals and resulting in systematic underestimation of true pathogen prevalence and transmission dynamics [8–10]. For other priority fungal pathogens acquired through environmental exposure, colonization biology is insufficiently characterized to predict non-clinical detectability [5].

Third, technical and economic constraints impede diagnostic advancement. As eukaryotes, fungal biology restricts selective drug targets and complicates assay development, while limited commercial markets for fungal diagnostics reduce industry investment incentives, leaving identification tools underdeveloped or inaccurate [6]. Inconsistent clinical reporting practices create systemic gaps in surveillance data even where diagnostic capacity exists. For instance, routine laboratory practice reports commensals without species-level identification, thereby masking the emergence of drug-resistant organisms and obscuring resistance emergence [10–12]. While nosocomial surveillance in high-income countries follows a hospital-based monitoring system and has access to advanced diagnostic tools like MALDI-TOF, such infrastructures are largely absent across low- and middle-income countries (LMICs), resulting in emergence of undetected genetic diversity and novel pathogenic lineages in these settings [12].

Wastewater-based surveillance offers a promising route to circumvent these structural limitations, as emerging evidence for pathogenic *Candida* species demonstrates. All colonised individuals, regardless of symptomatic status, healthcare utilisation, or diagnostic capacity, contribute biological material to municipal sewerage systems, revealing community-level pathogen circulation independent of clinical encounters [1].

## What does the evidence tell us about wastewater detection of fungal *pathogens*?

Published evidence on fungal WBS is currently limited to a small number of studies, mostly focused on *C. auris*, reflecting the nascent state of this field. *Candida* (*Candidozyma*) *auris* is a multidrug resistant fungal pathogen, first isolated from an ear infection in Japan in 2009 and now globally disseminated with high mortality rates [13]. A Southern Nevada surveillance study detected *C. auris* in 79% of wastewater samples collected over ten weeks from seven sewer-sheds, with elevated detection frequencies in areas serving healthcare facilities [8]. However, due to incomplete clinical case data and unknown human shedding rates, the statistical relationship between wastewater concentrations and clinical *C. auris* cases remains unverified [8].

Another surveillance study conducted in Florida [9] confirmed a 47% positivity rate for *C. auris* in hospital wastewater samples, compared to only 16% at downstream treatment plants, with concentrations lower at treatment sites. This dilution effect implies that sampling closer to points of high pathogen load, particularly hospitals, may yield stronger detection signals. Detection of patient-associated *C. auris* in ward sluice wastewater during an active UK hospital outbreak [14] demonstrated that unit-level sampling complements centralised municipal monitoring for nosocomial pathogens.

Published evidence establishes proof-of-concept but simultaneously exposes major gaps in fungal WBS. The biological distinction between yeasts and hyphal fungi implies no single surveillance framework captures all fungal pathogens equally. PCR-based detection identifies fungal nucleic acids but provides no information regarding infectivity, viability, or antimicrobial susceptibility profiles [15]—however, asymptomatic shedding for commensals increases background signals making presence/absence PCR analysis meaningless. Viability PCR addresses non-viable DNA amplification, and sequencing approaches help with strain-level discrimination between clinically relevant and environmental variants. Culture-based approaches enable strain characterisation but demonstrate poor sensitivity in wastewater matrices and cannot scale to population-level surveillance. Robust fungal WBS therefore requires parallel deployment of molecular and culture-based approaches for scalable community surveillance.

Recent work by Baker et al begins to extend this evidence base beyond *C. auris*, demonstrating simultaneous multiplex PCR detection of multiple WHO priority fungal pathogens in wastewater influents [15]. Quantifying asymptomatic *Candida* shedding will be essential to establish a relationship between clinical burden and wastewater concentrations; parallel clinical screening of defined populations and confirmed clinical case rates may offer proxy denominators for wastewater signal calibration in the interim [1]. WBS therefore functions as an early warning signal to guide and prioritise targeted clinical surveillance, particularly in non-endemic settings where the presence of pathogenic fungi may otherwise go undetected, and in resource-limited settings where individual diagnostic testing at scale is not feasible.

## Can fungi persist and spread through environmental water systems?

Beyond clinical settings, understanding whether fungal pathogens persist in environmental water systems is critical to assess the risk of environmentally acquired antifungal resistance [16]. *C. auris* has subsequently been isolated across various environments [11], such as remote coastal wetlands in India’s Andaman Islands, specifically from sandy beach and salt marsh sediments with minimal anthropogenic contact [17], suggesting a long-standing environmental presence predating clinical recognition. Viable organism recovery from harsh, oligotrophic environments indicates environmental adaptation rather than transient contamination.

Environmental antimicrobial resistance development represents an under-recognised and potentially high impact public health threat. While clinical resistance emerges through prolonged therapeutic exposure in individual patients, environmental resistance operates at population scale through distinct ecological and evolutionary mechanisms. Subinhibitory concentrations of antimicrobial pharmaceuticals enter aquatic ecosystems *via* wastewater effluent, CSO discharges, and agricultural application of contaminated biosolids [18]. This creates chronic, selective pressure across entire environmental fungal communities that may further enrich resistant pathogenic strains [19]. A study of Hong Kong mangrove ecosystems confirmed a high prevalence of *C. parapsilosis* demonstrating multidrug resistance to posaconazole, itraconazole, and amphotericin B, highlighting the emergence of the *C. parapsilosis* complex as a significant drug-resistant threat [20]. Critically, all multidrug-resistant isolates originated from sites around wastewater treatment facilities, suggesting a possible association between community antimicrobial consumption and environmental resistance.

**Fig 1:**
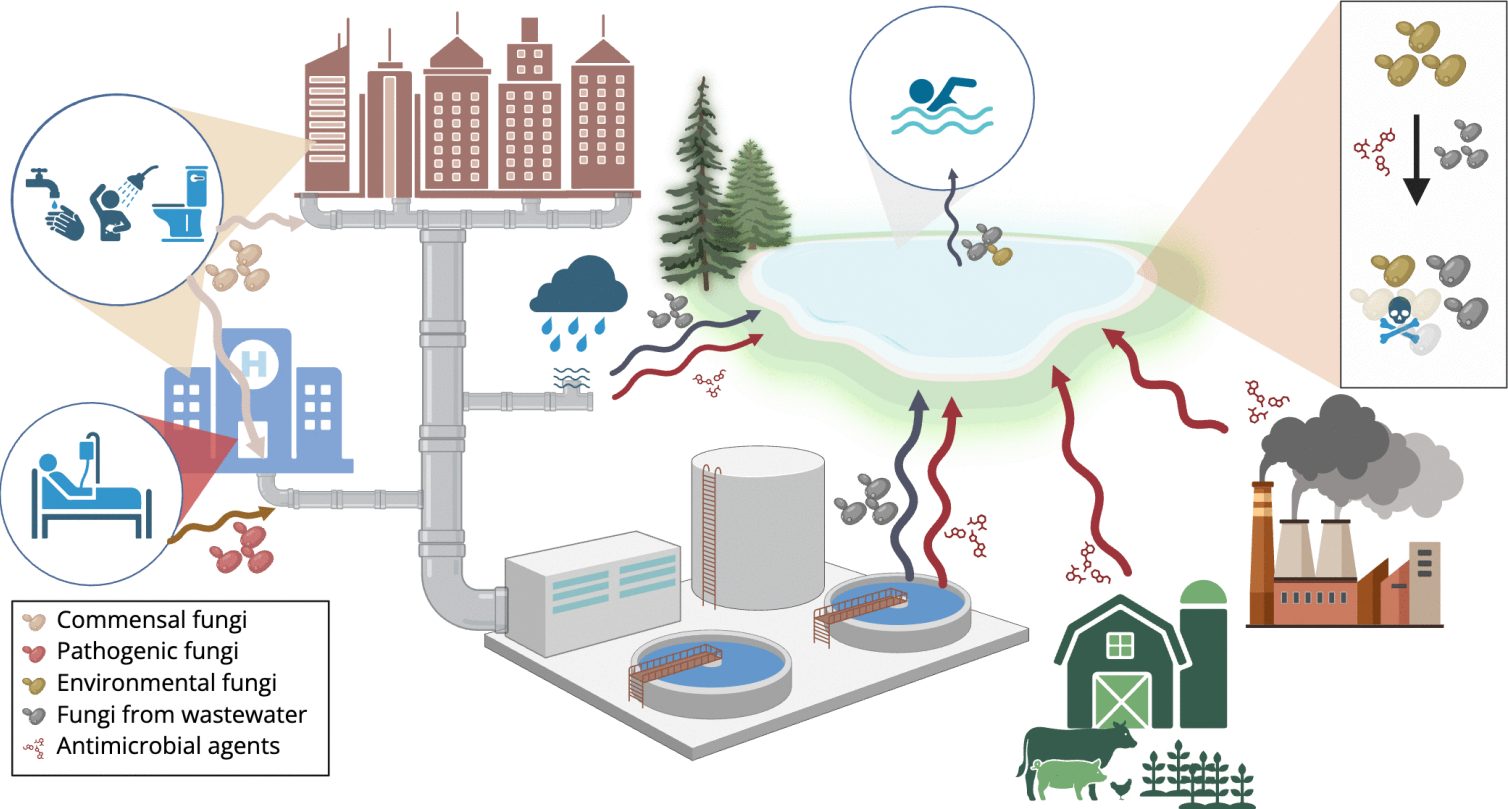
The role of wastewater in the environmental cycle of fungal pathogens and antifungal resistance. Fungal yeasts and moulds circulate between human, environmental, and agricultural reservoirs through multiple interconnected pathways. Commensal pathogenic fungi shed by hospital patients and community residents enter municipal sewerage systems, where wastewater-based epidemiological sampling enables population-scale surveillance of clinically relevant species. Wastewater treatment provides incomplete pathogen removal; during combined sewer overflow events, untreated effluent bypasses the treatment plant and discharges directly into receiving water bodies, introducing viable organisms into environmental reservoirs. Antifungal pharmaceutical residues enter aquatic ecosystems through treatment plant effluent, agricultural application of contaminated biosolids, and industrial discharge, thereby creating selective pressure across environmental fungal communities. This drives resistance acquisition in environmental fungal populations (inset, top right), which may subsequently colonise humans through recreational water contact or agricultural exposure. Arrows indicate direction of pathogen and antifungal agent movement; arrow colour corresponds to fungal type as indicated in the legend. Created in BioRender. Hepo-oja, P. (2026) https://BioRender.com/t7lvguf.

These results indicate that environmental fungi developing resistance through low-dose pharmaceutical exposure could colonise humans through recreational water contact, agricultural exposure, or food contamination. Once present in the community, within-household or person-to-person transmission represents an additional route of spread (Fig 1). Immunocompromised individuals colonised with resistant fungi of environmental origin could experience treatment failure despite no prior antimicrobial exposure. Unlike clinical resistance mechanisms tracked through hospital surveillance, environmental resistance pathways remain unmapped. Key unresolved questions include the concentrations of antifungal residues reaching receiving waters, the rates at which environmental fungi develop resistance under subinhibitory exposure, the role of biofilms, chromosomal instability, and horizontal gene transfer play in resistance amplification, and whether resistant strains can competitively displace susceptible populations. Understanding transmission pathways is essential for evidence-based intervention design, yet no systematic framework exists for investigating fungal ecology at the human-environment interface. While current evidence supports WBS as a tool for detecting commensal shedding, its potential to capture environmental fungal pathogen burden represents an unexplored surveillance function.

## What needs to happen next?

While current evidence is largely confined to *Candida* species, the WBS framework of integrating molecular detection, culturing, wastewater sampling and epidemiological signal interpretations is in principle applicable to other priority fungal pathogens as species-specific detection methods are validated. Key research domains that need to be critically addressed include:

- Standardisation of methodologies, including establishing optimal sampling locations, frequencies, and molecular detection platforms.
- Development of frameworks supporting translation of wastewater measurements into validated public health metrics that trigger interventions.
- Better understanding of human shedding of colonizing fungi into wastewater.
- Biological validation of an infection threshold, to determine what proportion of environmental fungi can establish human infections.
- Developing antifungal resistance surveillance plans to track emergence and dissemination of resistant strains.
- Predictive modelling to establish whether wastewater data can forecast clinical outbreaks.

Future surveillance should adopt a One Health framework, integrating wastewater-based epidemiology with clinical, veterinary, and agricultural monitoring to better capture the ecological drivers of antifungal resistance. As *C. auris* and other resistant fungi disseminate globally, the blind spot at the centre of public health infrastructure is not a technical failure. The tools exist, as COVID-19 surveillance demonstrated; instead, it is a failure of prioritisation. Wastewater-based surveillance offers the early standardisation that antimicrobial resistance monitoring lacked for decades and represents a scalable route to closing the infrastructure gap before the next fungal threat outpaces our capacity to respond.
